# Occupational Computer-Use Duration and Ulnar Nerve Electrodiagnostic Findings: An Exploratory Observational Study

**DOI:** 10.3390/biomedicines14081676

**Published:** 2026-07-25

**Authors:** Georgiana-Anca Vulpoi, Tudor-Ștefan Rotaru, Cătălina Elena Bistriceanu, Lenuța Bîrsanu, Codrina-Madalina Palade, Dan Iulian Cuciureanu

**Affiliations:** 1 Grigore T. Popa University of Medicine and Pharmacy Iasi, 700115 Iași, Romania; 2 Arcadia Hospitals and Medical Centers, 38 Sarariei Street, 700116 Iași, Romania; 3 Romania Department of Neurology, Elytis Hospital Hope, 43A Gheorghe Saulescu Street, 700010 Iasi, Romania; 4 “Socola” Institute of Psychiatry, 700282 Iasi, Romania; 5 Neurology Department I, “Prof. Dr. N. Oblu” Emergency Clinical Hospital, 2 Ateneului Street, 700309 Iasi, Romania

**Keywords:** peripheral nerves, nerve impairment, neurophysiology, ulnar neuropathy, computer use

## Abstract

**Background:** Musculoskeletal and peripheral nerve disorders have been reported among occupational computer users. However, the relationship between the duration of occupational computer use and ulnar neuropathy at the elbow (UNE) remains uncertain. This exploratory observational study investigated whether the duration of occupational computer use was associated with electrodiagnostically confirmed UNE and with ulnar nerve conduction parameters in a selected symptomatic population. **Methods:** Patients aged 23–68 years who were referred for nerve conduction studies (NCS) because of symptoms suggestive of upper-limb entrapment neuropathy were consecutively evaluated over a 1.5-year period. Among the 62 patients included, 22 met the neurophysiological diagnostic criteria for UNE, whereas 40 did not. The associations between occupational computer-use duration, bilateral ulnar sensory nerve action potential (SNAP) amplitudes, across-elbow motor nerve conduction velocities (NCV), and the presence of ulnar neuropathy were assessed using univariable and multivariable analyses. **Results:** Longer occupational computer-use duration showed modest unadjusted associations with lower bilateral ulnar SNAP amplitudes but was not associated with across-elbow motor NCV. In multivariable analysis, age and sex were significant independent predictors of SNAP amplitude, whereas computer-use duration was not (B = −0.086, β = −0.048, *p* = 0.756). Exposure duration was also not independently associated with bilateral changes in across-elbow motor NCV or with electrodiagnostically confirmed UNE. **Conclusions:** The findings do not support an independent association between the number of years of occupational computer use and ulnar nerve electrodiagnostic abnormalities beyond the effects of age and sex. However, small associations cannot be excluded because of the limited sample size, the indirect assessment of biomechanical exposure, and the inclusion of a selected symptomatic referral population.

## 1. Introduction

Current evidence suggests a potential association between prolonged computer use and the development of upper-limb compressive neuropathies [1]. However, most available studies have focused on carpal tunnel syndrome (CTS), whereas data on ulnar neuropathy at the elbow remain limited [2]. Cubital tunnel syndrome (CuTS), or ulnar neuropathy at the elbow, is generally regarded as the second most common entrapment neuropathy of the upper limb after CTS [3]. Epidemiological data remain limited; Roquelaure et al. reported a prevalence of clinically diagnosed CuTS of 0.6% in men and 0.8% in women in a French working population [4]. In a previous study including 148 symptomatic computer keyboard operators referred to an electrodiagnostic laboratory for suspected focal upper-limb neuropathies, focal ulnar neuropathy at the elbow (UNE) was identified in 74.5% of cases [5]. Pascarelli and Hsu reported clinical findings compatible with CuTS in 64% of a cohort of 485 patients with work-related upper-extremity symptoms, most of whom were computer users or musicians [6].

Previous ergonomic and epidemiological research has shown that computer-related work may be associated with upper-limb musculoskeletal disorders, particularly when prolonged exposure is combined with non-neutral postures, repetitive movements, static muscle loading, and suboptimal workstation design [7]. Sustained elbow flexion during computer work may increase pressure on the ulnar nerve at the elbow. Experimental pressure measurements have shown an increase from 0 to 19 mmHg in full extension to more than 200 mmHg during elbow flexion, supporting a plausible mechanism by which prolonged non-neutral elbow posture may contribute to chronic focal ulnar neuropathy [8].

UNE may result from local mechanical factors such as sustained elbow flexion, direct cubital tunnel pressure, and prolonged static upper-limb postures. Although computer work may involve these exposures, the number of years of computer use reflects only the duration of exposure, not the biomechanical load. Evidence regarding the relationship between the duration of computer use and electrodiagnostic confirmed UNE remains limited. It is unclear whether longer occupational computer use is associated with focal across-elbow conduction slowing or with changes in continuous ulnar nerve conduction measures.

Although local biomechanical factors may contribute to ulnar nerve compression at the elbow, these exposures were not directly assessed in the present study. We therefore hypothesised that the duration of occupational computer use might be associated with electrodiagnostic evidence of ulnar nerve impairment. The aim of this exploratory study was to investigate whether the duration of occupational computer use was associated with electrodiagnostics confirmed ulnar neuropathy at the elbow and with continuous ulnar nerve conduction measures in a selected symptomatic referral population undergoing evaluation for suspected upper-limb entrapment neuropathy.

## 2. Materials and Methods

### 2.1. Participants

Data were prospectively collected from consecutive patients referred to the electrodiagnostic laboratory for symptoms suggestive of compressive neuropathies of the upper limb, including tingling, numbness in the fingers or hand, and elbow pain. Among the 62 included participants, 22 presented symptoms suggestive of ulnar neuropathy at the elbow, whereas 40 presented predominant symptoms suggestive of CTS, without specific ulnar nerve-related manifestations. Therefore, the latter subgroup should not be regarded as a healthy control group but as a clinical subgroup without symptoms specifically attributable to the ulnar nerve. The study did not include a separate control group of healthy individuals without occupational exposure to computers. Neurophysiological examinations were performed by a neurologist specialising in clinical neurophysiology. The electrodiagnostic study was carried out using a 3-channel Litebox electromyograph (Neurosoft LLC, Ivanovo, Russia).

Patients were enrolled over an approximately 18-month period between 2024 and 2025. The inclusion criteria comprised the presence of the above-mentioned symptoms and an occupation involving computer-based work for more than three years, with mouse and keyboard use exceeding four hours per day, five days per week. Occupational computer exposure was assessed primarily according to the reported number of years of computer use. This variable was intended as a broad measure of cumulative exposure and did not quantify specific biomechanical factors such as daily hours of use, elbow flexion, direct pressure over the cubital tunnel, use of armrests, frequency of breaks, or workstation ergonomics. The exclusion criteria were pregnancy at the time of evaluation, a history of wrist or elbow trauma, and age under 18 years.

All examinations were performed in a temperature-controlled environment to maintain a skin temperature of 32 °C. The skin was cleaned before electrode placement to minimise potential grounding artefacts. All participants provided written informed consent before enrolment. The study was conducted in accordance with the Declaration of Helsinki, and the protocol was approved by the Ethics Committee of the “Grigore T. Popa” University of Medicine and Pharmacy, Iasi, Romania.

Collected data were recorded in an Excel database and included age, sex, hand dominance, number of years of computer use, profession, comorbidities, and electrophysiological parameters. Medical history was also reviewed to exclude other known neuromuscular conditions, such as cervical radiculopathy, brachial plexopathy, or prior hand surgery. All data were anonymized before analysis.

A total of 62 participants who met the predefined inclusion and exclusion criteria were included in the study. Regarding hand dominance, only one participant reported left-hand dominance, while the remaining participants were right-handed. Hand numbness was not associated with cervical pain. None of the participants reported sensory symptoms in the lower limbs, and the onset of hand symptoms was not preceded by distal lower-limb symptoms. Furthermore, the sensory complaints followed a focal distribution consistent with median or ulnar nerve involvement rather than a symmetric, length-dependent pattern. Therefore, a clinically manifest distal symmetric polyneuropathy was considered unlikely.

The computer user group included IT programmers, teachers who used computers in their daily activities, accountants, and secretarial staff. These participants had no history of upper-limb surgical interventions or traumatic injuries and no other known musculoskeletal disorders of the upper limbs.

### 2.2. Neurophysiological Testing

For the neurophysiological assessment, single-use surface electrodes were used. The nerve conduction studies (NCS) included both sensory and motor assessment of the median and ulnar nerves. However, in the present study, only the electrophysiological parameters of the ulnar nerve were analysed.

For the ulnar motor NCS, the recording electrode was placed over the abductor digiti minimi muscle belly. Electrical stimulation was delivered at the wrist, 7–8 cm proximal to the recording electrode, and below and above the elbow. For median motor NCS, the recording electrode was placed over the abductor pollicis brevis muscle. The median nerve was stimulated at the wrist and cubital fossa, 7–8 cm proximal to the recording electrode, between the palmaris longus and flexor carpi radialis tendons.

Sensory responses were obtained using antidromic stimulation at the wrist. Median nerve sensory responses were recorded from the third digit, with a distance of 13–14 cm between the stimulation and recording sites. Ulnar nerve sensory responses were recorded from the fifth digit, with a distance of 10–12 cm between the stimulation and recording sites.

Electrodiagnostic criteria were interpreted in accordance with the American Association of Neuromuscular & Electrodiagnostic Medicine (AANEM) practice parameter for ulnar neuropathy at the elbow. The AANEM considers an across-elbow motor nerve conduction velocity below 50 m/s, or a slowing of more than 10 m/s relative to the forearm segment, to be suggestive of a focal ulnar nerve lesion at the elbow [9]. Electrophysiological severity was then graded according to the Padua neurophysiological classification for ulnar nerve entrapment across the elbow. This classification stratifies cases based on motor conduction slowing across the elbow, ulnar sensory nerve action potential amplitude, and the presence or absence of sensory and motor evoked responses [10]. Of the 62 included participants, 22 fulfilled the Padua electrodiagnostic criteria for CuTS. Among these 22 patients, 14 patients (63.6%) had mild involvement, defined as slowing of motor conduction velocity across the elbow with a normal ulnar sensory nerve action potential. Eight patients (36.4%) had moderate involvement, defined by slowing of motor conduction velocity across the elbow associated with reduced ulnar sensory nerve action potential amplitude. No severe cases, defined by slowing of motor conduction velocity across the elbow with absent ulnar sensory nerve action potential, and no extreme cases, defined by absent ulnar sensory nerve action potential and absent compound muscle action potential recorded from the abductor digiti minimi, were identified in this cohort.

Regarding the side of involvement, 15 patients (68.2%) had isolated left ulnar nerve involvement, 4 patients (18.2%) had isolated right ulnar nerve involvement, and 3 patients (13.6%) had bilateral ulnar nerve involvement.

Statistical analysis was performed using IBM SPSS Statistics, version 20.0, and effect size was calculated using G*Power 3.1.9.7. Analyses were conducted using available case data. Participants with missing values for one or more variables were excluded only from the analysis involving those variables, and no imputation of missing data was performed. Consequently, the sample size varied across analyses. The number of observations included in each analysis is reported separately.

Quantitative variables were expressed as median and interquartile range or as mean and standard deviation, depending on data distribution, while qualitative variables were presented as absolute frequencies and percentages. Due to the small sample size, nonparametric tests and nonparametric correlations were used.

Paired comparisons between right and left upper-limb values were performed using the Wilcoxon signed-rank test. Associations between age, exposure duration, and neurophysiological parameters were assessed using Spearman’s correlation coefficient. The relationship between exposure categories and the presence of ulnar neuropathy was analysed using tests for categorical variables, with cautious interpretation in cases with low frequencies. The threshold for statistical significance was set at *p* < 0.05, and all tests were two-tailed. Given the exploratory nature of the study, no formal adjustment for multiple comparisons was applied. Accordingly, *p*-values were interpreted cautiously, and statistically significant findings were considered hypothesis-generating rather than confirmatory. Where applicable, effect estimates were reported with 95% confidence intervals to indicate their precision and potential clinical relevance.

## 3. Results

The study cohort comprised 62 patients (25 men and 37 women). The mean age was 46.53 years (SD = 11.92), with an age range of 23 to 68 years. The mean duration of arm exposure was 18.21 years (SD = 8.54), ranging from 2 to 40 years. Only 22 patients met the defined criteria for cubital tunnel syndrome.

For all patients included in the study, a significant difference exists between the median SNAP amplitude for the ulnar nerve in the right and left arms (28.2 vs. 24.5 µV; Wilcoxon signed-rank test: Z=−2.85, p<0.01).

**Figure 1:**
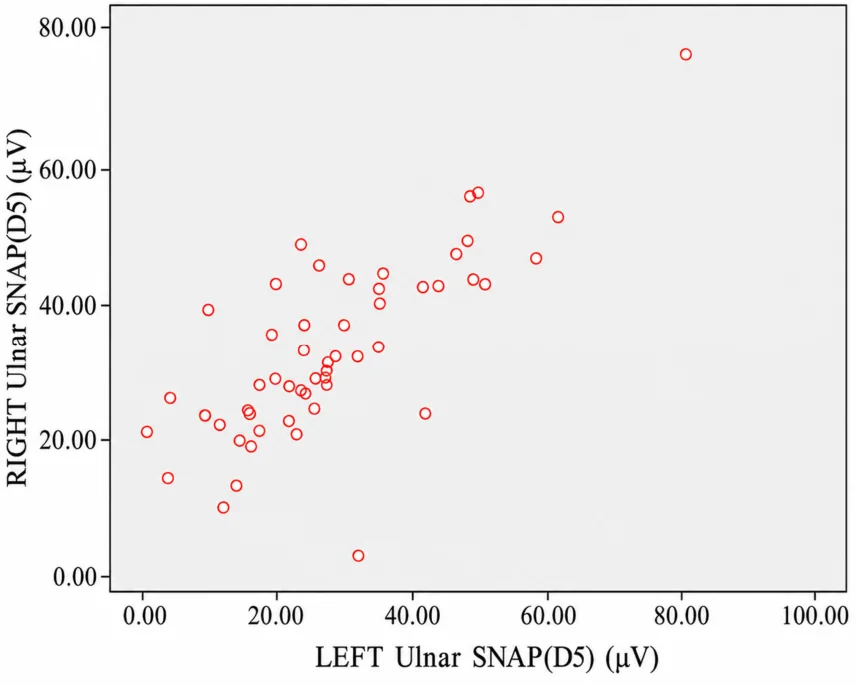
Relationship between right and left ulnar sensory nerve action potential amplitudes. The scatter plot shows a strong positive bilateral correlation (r = 0.780, *p* < 0.001; n = 34). SNAP, sensory nerve action potential.

A strong positive correlation was observed between right and left ulnar SNAP amplitudes among participants with available bilateral measurements (r = 0.780, *p* < 0.001; n = 34) (Figure 1). Higher left-sided SNAP amplitudes were generally associated with higher right-sided amplitudes, indicating substantial bilateral concordance, although some individual variability was present.

**Figure 2:**
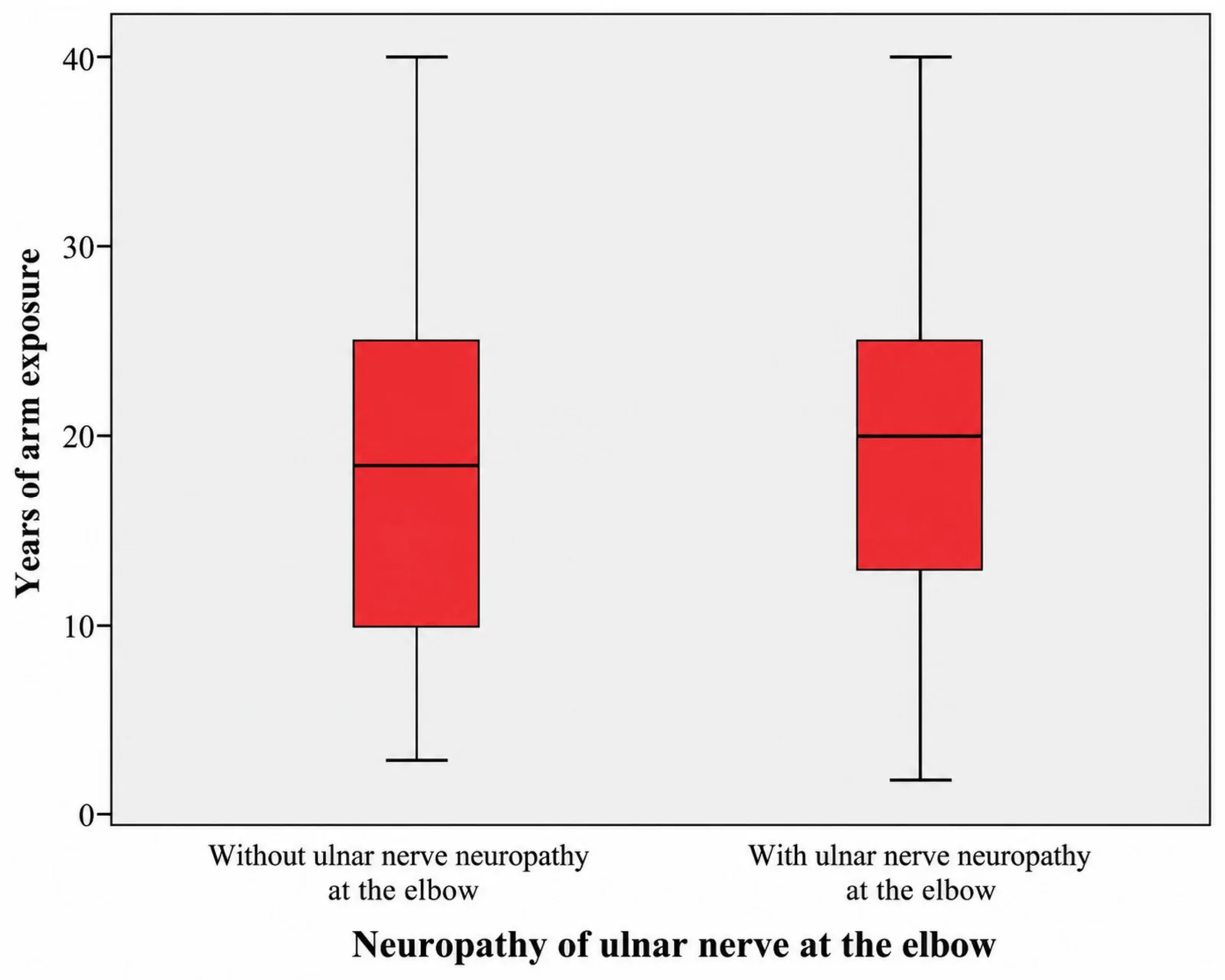
Distribution of occupational computer-use duration according to the presence or absence of electrodiagnostically confirmed ulnar neuropathy at the elbow. The boxplots represent the median, interquartile range, and observed range.

The distribution of occupational computer-use duration showed substantial overlap between patients with and without electrodiagnostically confirmed ulnar neuropathy at the elbow (Figure 2). Although the median duration was slightly higher in patients with neuropathy, the visual difference was small, and considerable variability was observed in both groups. In the primary inferential analyses, exposure duration was evaluated as a continuous variable rather than through predefined categories. Occupational computer-use duration, analyzed as a continuous variable, was not retained in the final multivariable model for the presence of ulnar neuropathy at the elbow.

As shown in Table 1, Spearman’s correlation analysis revealed a strong positive association between patient age and duration of occupational computer use (ρ = 0.715; *p* < 0.001). Both age and exposure duration were negatively correlated with bilateral ulnar SNAP amplitudes. Age showed moderate negative correlations with right SNAP (ρ = −0.508; *p* = 0.001) and left SNAP (ρ = −0.576; *p* < 0.001), while exposure duration was negatively correlated with right SNAP (ρ = −0.354; *p* = 0.027) and left SNAP (ρ = −0.395; *p* = 0.019). Neither age nor exposure duration was significantly associated with right or left across-elbow NCV. Among participants with available bilateral measurements, right and left SNAP amplitudes were strongly correlated (ρ = 0.823; *p* < 0.001; n = 34), while right and left NCV values showed a moderate positive correlation (ρ = 0.588; *p* = 0.003; n = 23).

As shown in Table 2, Spearman’s correlation analysis revealed a strong positive association between patient age and years of occupational computer use (ρ = 0.728; *p* < 0.001). Patient age was negatively correlated with both right and left ulnar SNAP amplitudes. Similarly, longer occupational computer-use duration was associated with lower bilateral ulnar SNAP amplitudes, whereas no significant associations were observed with right or left across-elbow NCV. However, these findings represent unadjusted associations and may be confounded by age. Therefore, age-adjusted partial correlation analyses were subsequently performed (Table 3).

In the overall study sample with available bilateral measurements, right and left ulnar SNAP values were strongly correlated (r = 0.739; *p* < 0.001), indicating bilateral concordance in sensory amplitudes, as illustrated in Figure 1. A weak positive correlation was also observed between right ulnar SNAP and right ulnar nerve conduction velocity at the elbow (r = 0.327; *p* = 0.035), while right and left ulnar nerve conduction velocities showed a moderate positive correlation (r = 0.576; *p* < 0.001).

In the partial correlation analysis, after controlling patient age, arm exposure duration was not significantly correlated with left ulnar nerve SNAP values (r = −0.150; *p* = 0.278) or right ulnar nerve SNAP values (r = −0.027; *p* = 0.845) (Table 3). In contrast, left and right ulnar nerve SNAP values remained strongly correlated (r = 0.712; *p* < 0.001), suggesting bilateral concordance in sensory amplitudes independent of age.

In patients who met the criteria for CuTS, years of exposure were negatively correlated with bilateral SNAP values, as shown in Table 4. This correlation was observed for both right (r = −0.587; *p* = 0.004) and left (r = −0.536; *p* = 0.012) SNAP, suggesting a reduction in sensory amplitudes with increasing exposure duration. A negative correlation was also observed between exposure duration and ulnar nerve conduction velocity at the elbow on the right side (r = −0.530; *p* = 0.042), whereas the corresponding association on the left side did not reach statistical significance. These results should be interpreted with caution, given the small size of the analysed subgroup and the strong correlation between age and exposure duration.

Causality cannot be inferred based solely on these correlations. Therefore, age-controlled partial correlations were computed to assess whether the relationship between exposure duration and neurophysiological parameters remained significant after accounting for age (Table 5). After controlling for patient age, arm exposure duration was no longer significantly correlated with right ulnar nerve SNAP values (r = 0.004; *p* = 0.991), left ulnar nerve SNAP values (r = −0.167; *p* = 0.623), right ulnar nerve conduction velocity at the elbow (r = −0.255; *p* = 0.449), or left ulnar nerve conduction velocity at the elbow (r = −0.236; *p* = 0.485). The only significant association remaining was the positive correlation between right and left ulnar nerve SNAP values (r = 0.688; *p* = 0.019), suggesting bilateral concordance in sensory amplitudes independent of age.

We also performed a multiple regression analysis using the Stepwise method (Table 6 and Table 7), with the SNAP score for the left hand as the dependent variable and the following as independent variables: patient age, patient sex, number of years of arm exposure, dominant hand (right or left), and the presence or absence of ulnar nerve neuropathy at the elbow. The results are similar for the left hand. There are only two significant predictors of the left-hand SNAP score, which together explain 42.8% of the variance in the dependent variable (adjusted R^2^ = 0.428). The first (negative) predictor is the patient’s age (β = −0.619; *p* < 0.05), and the second predictor is the patient’s sex (β = 0.558; *p* < 0.05). Female gender and younger age significantly explain approximately 43% of the variance in the left-hand SNAP variable.

In the stepwise multiple linear regression analysis (Table 6), age entered the model at the first step and explained 23.5% of the variance in right ulnar SNAP amplitude (adjusted R^2^ = 0.222). Sex was added at the second step, increasing the explained variance to 44.9% (adjusted R^2^ = 0.430). The remaining candidate variables were not retained in the final model.

In the final stepwise regression model (Table 7), increasing age was independently associated with lower right ulnar SNAP amplitude (B = −0.801 µV per year; standardised β = −0.642; *p* < 0.001). Female sex was independently associated with a 14.522 µV higher right ulnar SNAP amplitude compared with male sex, after adjustment for age (B = 14.522; standardised β = 0.490; *p* < 0.001).

In the stepwise multiple linear regression analysis (Table 8), age entered the model at the first step and explained 17.7% of the variance in left ulnar SNAP amplitude (adjusted R^2^ = 0.162). Sex was added at the second step, increasing the proportion of explained variance to 44.9% (adjusted R^2^ = 0.428). The remaining candidate variables were not retained in the final model.

In the final stepwise regression model for left ulnar SNAP amplitude (Table 9), increasing age was independently associated with lower SNAP values (B = −0.804 µV per year; standardised β = −0.619; *p* < 0.001). Female sex was independently associated with a 17.899 µV higher left ulnar SNAP amplitude compared with male sex, after adjustment for age (B = 17.899; standardised β = 0.558; *p* < 0.001). Both age and sex therefore remained significant predictors in the final model.

Multicollinearity among the predictors included in the logistic regression model was assessed using tolerance and variance inflation factor values (Table 10). The VIF was 2.33 for the duration of occupational computer use and 3.43 for age, with corresponding tolerance values of 0.43 and 0.29, respectively. These values indicate a moderate degree of shared variance between age and exposure duration but do not suggest severe multicollinearity. Therefore, both variables could be retained in the model, although their individual effects should be interpreted cautiously.

A multiple linear regression model was performed using right ulnar SNAP amplitude as the dependent variable, with age, sex, hand dominance, occupational computer-use duration, and electrodiagnostically confirmed ulnar neuropathy at the elbow entered simultaneously (Table 11 and Table 12). The model explained 49.1% of the variance in SNAP amplitude ($R^{2}=0.491$; adjusted $R^{2}=0.440$).

Older age was independently associated with lower SNAP amplitude (B=−0.741, standardized β=−0.570, p=0.001), whereas female sex was associated with higher SNAP amplitude (B=14.947, standardized β=0.466, p<0.001). Occupational computer-use duration was not independently associated with SNAP amplitude (B=−0.086, standardized β=−0.048, p=0.756). Hand dominance (B=−12.669, p=0.303) and the presence of ulnar neuropathy at the elbow (B=−6.447, p=0.094) were also not statistically significant predictors.

Separate multiple linear regression models were performed for right and left across-elbow motor nerve conduction velocity, with age, sex, and occupational computer-use duration entered simultaneously (Table 13 and Table 14). Occupational computer-use duration was not independently associated with right-sided conduction velocity (B=0.355, standardized β=0.155, p=0.522) or left-sided conduction velocity (B=−0.279, standardized β=−0.138, p=0.547). Age and sex were also not statistically significant predictors in either model.

Collinearity diagnostics showed no evidence of severe multicollinearity among the predictors. Across the models, VIF values ranged from 1.025 to 2.543, while tolerance values ranged from 0.393 to 0.976. These values indicate low-to-moderate collinearity and support the simultaneous inclusion of age, sex, and occupational computer-use duration in the regression models.

The multiple linear regression model explained 45.7% of the variance in right ulnar SNAP amplitude (R^2^ = 0.457; adjusted R^2^ = 0.408) and 49.1% of the variance in left ulnar SNAP amplitude (R^2^ = 0.491; adjusted R^2^ = 0.440) (Table 15 and Table 16).

In the exploratory binary logistic regression analysis evaluating the presence of UNE, occupational computer-use duration was not a significant predictor, whereas male sex was the only variable retained as a significant predictor in the final model.

The recorded comorbidities included diabetes mellitus, obesity, rheumatic diseases, and hypothyroidism. Comorbidities were available only as an aggregate count: 44 participants (71.0%) had no known comorbidities, 15 (24.2%) had one, and 3 (4.8%) had two comorbidities.

## 4. Discussion

Previous studies have evaluated the relationship between computer use and upper-limb peripheral nerve disorders, most commonly focusing on CTS or sensory ulnar NCS changes at the wrist. Only a limited number have specifically addressed ulnar neuropathy at the elbow using neurophysiological criteria.

Data from the literature suggest a possible association between computer use and subtle neurophysiological changes in the peripheral nerves of the upper limb. Thus, a neurography study in office workers with and without neck pain showed that 1 h of computer use may delay ulnar nerve sensory NCV, particularly in participants with cervical symptoms [11]. Similarly, another study conducted on 21 male computer users and 21 male control subjects reported significantly reduced sensory NCVs of the median and ulnar nerves at the wrist bilaterally, in asymptomatic office workers compared with non-computer users [12].

The main finding of the present study was that the duration of occupational computer use was not independently associated with electrodiagnostically confirmed ulnar neuropathy or with the analysed ulnar nerve conduction parameters. The substantial overlap in exposure duration between patients with and without ulnar neuropathy further indicates that years of computer use alone do not clearly distinguish the two groups. However, cumulative duration is an indirect measure of exposure and does not capture potentially relevant biomechanical factors, such as prolonged elbow flexion, direct cubital pressure, inadequate arm support, repetitive movements, or sustained postures. Therefore, the present findings do not exclude a possible contribution from specific computer-related ergonomic behaviours that were not directly assessed.

Right and left ulnar SNAP amplitudes were strongly correlated, suggesting the influence of shared individual and technical factors, particularly age and sex. This bilateral concordance should not be interpreted as evidence of bilateral neuropathy or an occupational effect. Although SNAP amplitudes were lower in the left upper limb, the magnitude of the difference was limited. This asymmetry could hypothetically reflect unmeasured postural or local mechanical factors; however, these exposures were not evaluated directly.

These findings should be interpreted in the context of a selected symptomatic referral population and should not be generalised to asymptomatic occupational computer users. The limited sample size and indirect exposure assessment also mean that small, nonlinear, or subgroup-specific associations cannot be excluded.

The duration of occupational computer use, expressed in years, does not directly reflect the intensity of biomechanical exposure. In a case–referent study, occupational activities involving greater force were associated with electrodiagnostically confirmed ulnar neuropathy, particularly in the presence of non-neutral joint postures, whereas repetitive movements considered independently were not significantly associated with confirmed neuropathy [13]. In another case–referent study, daily computer-use duration was not significantly associated with ulnar neuropathy, whereas resting the elbow on the worktable for at least two hours per day was associated with a higher likelihood of electrodiagnostically confirmed ulnar neuropathy (OR = 2.16; 95% CI, 1.06–4.44) [14]. These findings suggest that local mechanical loading and workstation-related behaviours may be more relevant than cumulative computer-use duration alone. Additional unmeasured factors, including workstation ergonomics, daily exposure patterns, prolonged elbow flexion, local contact pressure, rest-break frequency, individual characteristics, comorbidities, and other occupational or recreational activities, may also influence susceptibility to ulnar nerve compression. Evidence that specific rest-break schedules prevent electrodiagnostically confirmed UNE remains limited, although a Cochrane review found very low-certainty evidence that additional breaks may modestly reduce upper-limb discomfort [15]. Because these factors were not assessed in the present study, residual confounding cannot be excluded.

Although years of occupational computer use were associated with lower bilateral ulnar SNAP amplitudes in the unadjusted analysis, this association was no longer significant after adjustment for age. The strong correlation between age and exposure duration suggests that age may have confounded the apparent relationship between cumulative exposure and sensory nerve responses. This interpretation is consistent with previous evidence showing an age-related decline in sensory nerve action potential amplitudes, including those of the ulnar nerve. In a longitudinal study of working adults, Tong et al. reported a gradual decrease in ulnar SNAP amplitude, mild prolongation of sensory latencies, and a slight reduction in sensory conduction velocity over a 5-year follow-up period [16]. In this context, the unadjusted association between longer occupational computer-use duration and lower bilateral ulnar SNAP amplitudes should be interpreted cautiously, as age appears to be an important confounding factor in this selected symptomatic population.

Separate multiple linear regression models were performed for right and left ulnar SNAP amplitudes. Age and sex were the only predictors retained, with the models explaining 44.9% of the variance in right SNAP amplitude (adjusted R^2^ = 0.430) and 44.9% of the variance in left SNAP amplitude (adjusted R^2^ = 0.428). Older age was associated with lower SNAP amplitudes, whereas female sex was associated with higher values. Hand dominance, electrodiagnostically confirmed UNE, and occupational computer-use duration were not retained in the final models. Given the limited sample size and exploratory nature of the analyses, however, a small occupational effect cannot be excluded.

Ulnar SNAP amplitude is a relatively nonspecific neurophysiological measure that may be influenced by age, sex, interindividual anatomical variability, axonal disorders of different etiologies, and technical recording factors. Although reduced SNAP amplitude may indicate sensory axonal involvement, it does not independently localise the lesion to the elbow and should not be interpreted as specific evidence of occupational ulnar nerve injury.

The interpretation of occupational exposure was also limited by its assessment solely as the number of years of computer use. This measure does not account for daily exposure intensity, elbow position, direct pressure over the cubital tunnel, upper-limb posture, workstation configuration, or the frequency of breaks. Thus, individuals with similar exposure durations may have substantially different biomechanical loads on the ulnar nerve.

Collinearity diagnostics showed no evidence of severe multicollinearity among the variables included in the models. Similarly, exploratory logistic regression did not identify occupational computer-use duration as an independent predictor of electrodiagnostically confirmed ulnar neuropathy. Together with the absence of a consistent association with NCV and of a clear exposure–severity gradient, these findings do not support a causal or dose-dependent relationship. Rather, they suggest that any potential effect of occupational computer use, if present, may depend on individual susceptibility and on specific biomechanical exposures not captured by cumulative duration alone. In this context, occupational exposure may represent a possible contributing or precipitating factor in predisposed individuals, although this hypothesis was not directly tested in the present study [17].

The modest associations observed in the unadjusted analyses should be considered exploratory, particularly because multiple comparisons were performed without formal adjustment. Their interpretation should therefore emphasise effect size and precision rather than statistical significance alone, and confirmation in larger prospective studies is warranted. Notably, a previous epidemiological study examining computer work as a potential risk factor for ulnar neuropathy reported an inverse exposure–response relationship between daily computer use and both electrodiagnostic abnormalities and ulnar neuropathy-like symptoms. In contrast, leaning on the elbow while working was suggested as a possible specific causal factor for ulnar neuropathy at the elbow, probably through direct pressure on the ulnar nerve in the elbow region [14].

The limited evidence regarding computer work and ulnar neuropathy at the elbow may partly reflect the multifactorial nature of the condition and the methodological difficulty of isolating relevant occupational risk factors. From a biomechanical perspective, ulnar neuropathy at the elbow is a dynamic and multifactorial condition. Elbow flexion alters cubital tunnel geometry and increases local pressure, while upper-limb movement requires longitudinal excursion and deformation of the ulnar nerve. Dynamic instability, including subluxation or dislocation over the medial epicondyle, may further expose the nerve to repetitive friction and external compression [18]. However, these mechanisms depend on the intensity and characteristics of the mechanical exposure rather than solely on its duration [19].

In the present study, occupational exposure was represented solely by the number of years of computer use, an indirect measure that does not capture the biomechanical characteristics most relevant to ulnar nerve compression. Therefore, the absence of an association with exposure duration should be interpreted narrowly and does not exclude a potential contribution from specific ergonomic or local mechanical factors that were not directly assessed. Accordingly, the lack of an independent association after adjustment for age and sex should not be regarded as evidence that no underlying biological relationship exists. It indicates only that, within this selected symptomatic sample, the cumulative duration of occupational computer use did not provide additional explanatory value beyond these covariates. Given the limited sample size and the indirect assessment of exposure, small effects or associations restricted to specific ergonomic contexts may have remained undetected. These findings should therefore be considered exploratory and hypothesis-generating and require confirmation in larger prospective studies incorporating objective measures of exposure intensity, upper-limb posture, sustained elbow flexion, local pressure, and workstation ergonomics.

### Strengths and Limitations

Nevertheless, several important limitations of the present study should be acknowledged. A major limitation of this study is the referral-based selection of participants. All patients were evaluated in a neurophysiology laboratory because of upper-limb symptoms suggestive of an entrapment neuropathy. Consequently, the study population differs substantially from a healthy occupational cohort, introducing selection bias and limiting the generalizability of the findings. Although patients without ulnar symptoms were analysed to explore possible subclinical ulnar nerve changes, they cannot be considered healthy controls because they presented symptoms suggestive of another entrapment neuropathy, predominantly CTS. Therefore, the results apply only to this selected clinical population and should not be extrapolated to occupational computer users in the general population.

A major limitation is the absence of an appropriate occupational control group without computer exposure. Consequently, the present study cannot determine whether occupational computer work itself is associated with a higher prevalence or risk of ulnar neuropathy compared with non-computer-based employment. The findings are limited to the relationship between the reported duration of computer use and ulnar nerve parameters within a selected symptomatic clinical population.

One conceptual limitation is that occupational computer use was quantified solely by years of use, a temporal measure that does not adequately capture biomechanical exposure. The observational design does not allow a direct causal relationship to be established between the duration of occupational computer exposure and UNE. Moreover, exposure duration was expressed in years, without detailed quantification of relevant ergonomic and biomechanical characteristics, such as daily duration of computer use, elbow contact with the working surface, upper-limb posture, or breaks during work. Consequently, the study did not directly assess the biomechanical mechanisms of ulnar nerve compression and cannot determine whether specific ergonomic factors are associated with neuropathy. Another limitation is the potential for residual confounding, as relevant occupational, ergonomic, lifestyle, and recreational exposures were not systematically documented. Their omission prevented statistical adjustment and limits both causal inference and assessment of the independent association between occupational computer use and ulnar nerve impairment.

However, because NCS of the lower limbs were not routinely performed in clinically unaffected territories, a subclinical polyneuropathy could not be definitively excluded.

The availability of electrodiagnostic measurements differed among participants and between sides, resulting in variable sample sizes across analyses. The use of available case analysis reduced the statistical power of some comparisons, particularly those involving across-elbow conduction velocity. In addition, bias due to missing data cannot be ruled out because the assumption that the data were missing completely at random could not be verified. Additionally, the small sample size, particularly in the subgroup and partial-correlation analyses, may have limited the statistical power to detect small or moderate associations. The relatively small number of participants with electrodiagnostically confirmed UNE further reduced the precision of the subgroup estimates. Accordingly, these findings should be interpreted cautiously as exploratory and require confirmation in larger studies. Therefore, non-significant findings should not be interpreted as evidence of no association, and these analyses should be regarded as exploratory. Furthermore, the absence of ulnar nerve ultrasonography precluded assessment of nerve enlargement and dynamic instability. Accordingly, the study characterises only electrodiagnostic abnormalities and cannot address the structural or dynamic mechanisms of ulnar nerve involvement. Comorbidities were recorded only as an aggregate count, preventing diagnosis-specific adjustment, while BMI, smoking, and alcohol data were unavailable; therefore, residual confounding cannot be excluded.

## 5. Conclusions

Within the limitations of this study, the findings do not support an association between the number of years of occupational computer use and electrodiagnostically confirmed ulnar neuropathy at the elbow in this selected symptomatic population. Age and sex were more strongly associated with ulnar SNAP amplitudes, whereas exposure duration was not an independent predictor and was not associated with focal across-elbow motor conduction slowing. Reduced SNAP amplitudes should therefore not be interpreted as specific evidence of focal or occupational ulnar nerve injury. The number of years of computer use represents only a temporal measure and does not adequately characterise biomechanical exposure. Accordingly, the results do not exclude a possible contribution of specific ergonomic or local mechanical factors, such as sustained elbow flexion or prolonged external compression, which were not directly assessed. The findings should not be extrapolated to healthy occupational computer users. Prospective studies involving representative occupational cohorts should incorporate detailed measures of daily computer use, upper-limb posture, elbow support, workstation ergonomics, and multimodal nerve assessment.

## Figures and Tables

**Table 1:** Spearman’s correlation results in patients without ulnar neuropathy at the elbow.

Variables	Spearman’s *ρ*	*p*	*n*
Patient age—years of arm exposure	0.715 **	<0.001	40
Patient age—right ulnar SNAP	−0.508 **	0.001	39
Patient age—left ulnar SNAP	−0.576 **	<0.001	35
Patient age—right ulnar NCV	−0.089	0.657	27
Patient age—left ulnar NCV	0.044	0.842	23
Years of arm exposure—right ulnar SNAP	−0.354 *	0.027	39
Years of arm exposure—left ulnar SNAP	−0.395 *	0.019	35
Years of arm exposure—right ulnar NCV	0.140	0.487	27
Years of arm exposure—left ulnar NCV	0.072	0.742	23
Right ulnar SNAP—left ulnar SNAP	0.823 **	<0.001	34
Right ulnar SNAP—right ulnar NCV	0.065	0.748	27
Right ulnar SNAP—left ulnar NCV	−0.258	0.234	23
Left ulnar SNAP—right ulnar NCV	−0.097	0.659	23
Left ulnar SNAP—left ulnar NCV	−0.250	0.250	23
Right ulnar NCV—left ulnar NCV	0.588 **	0.003	23

Note: SNAP, sensory nerve action potential recorded from digit 5; NCV, ulnar motor nerve conduction velocity across the elbow, recorded from the abductor digiti minimi muscle. * *p* < 0.05; ** *p* < 0.01 (two-tailed).

**Table 2:** Spearman’s correlation results in all patients.

Variables	Spearman’s *ρ*	*p*	*n*
Patient age—years of arm exposure	0.728 **	<0.001	62
Patient age—right ulnar SNAP	−0.443 **	<0.001	61
Patient age—left ulnar SNAP	−0.380 **	0.004	56
Patient age—right ulnar NCV	−0.085	0.594	42
Patient age—left ulnar NCV	0.090	0.566	43
Years of arm exposure—right ulnar SNAP	−0.422 **	0.001	61
Years of arm exposure—left ulnar SNAP	−0.429 **	0.001	56
Years of arm exposure—right ulnar NCV	−0.054	0.733	42
Years of arm exposure—left ulnar NCV	−0.077	0.622	43
Right ulnar SNAP—left ulnar SNAP	0.739 **	<0.001	55
Right ulnar SNAP—right ulnar NCV	0.327 *	0.035	42
Right ulnar SNAP—left ulnar NCV	0.161	0.303	43
Left ulnar SNAP—right ulnar NCV	0.022	0.896	37
Left ulnar SNAP—left ulnar NCV	0.262	0.094	42
Right ulnar NCV—left ulnar NCV	0.576 **	<0.001	36

Note: SNAP, sensory nerve action potential recorded from digit 5; NCV, ulnar motor nerve conduction velocity across the elbow, recorded from the abductor digiti minimi muscle. * *p* < 0.05; ** *p* < 0.01 (two-tailed).

**Table 3:** Partial correlations between exposure duration and ulnar SNAP amplitudes, adjusted for age.

Variables	Partial *r*	*p*	df
Exposure duration—left ulnar SNAP	−0.150	0.278	52
Exposure duration—right ulnar SNAP	−0.027	0.845	52
Left ulnar SNAP—right ulnar SNAP	0.712	<0.001	52

**Table 4:** Spearman’s correlation results in patients with ulnar nerve neuropathy at the elbow.

Variables	Spearman’s ρ	*p*	*n*
Patient age—years of arm exposure	0.770 **	<0.001	22
Patient age—right ulnar SNAP	−0.597 **	0.003	22
Patient age—left ulnar SNAP	−0.388	0.082	21
Patient age—right ulnar NCV	−0.283	0.306	15
Patient age—left ulnar NCV	−0.245	0.297	20
Years of arm exposure—right ulnar SNAP	−0.587 **	0.004	22
Years of arm exposure—left ulnar SNAP	−0.536 *	0.012	21
Years of arm exposure—right ulnar NCV	−0.530 *	0.042	15
Years of arm exposure—left ulnar NCV	−0.403	0.078	20
Right ulnar SNAP—left ulnar SNAP	0.552 **	0.009	21
Right ulnar SNAP—right ulnar NCV	0.736 **	0.002	15
Right ulnar SNAP—left ulnar NCV	0.345	0.136	20
Left ulnar SNAP—right ulnar NCV	0.029	0.923	14
Left ulnar SNAP—left ulnar NCV	0.364	0.125	19
Right ulnar NCV—left ulnar NCV	0.437	0.135	13

Note: SNAP, sensory nerve action potential recorded from digit 5; NCV, ulnar motor nerve conduction velocity across the elbow, recorded from the abductor digiti minimi muscle. * *p* < 0.05; ** *p* < 0.01 (two-tailed).

**Table 5:** Pearson partial correlation results for patients with ulnar nerve neuropathy at the elbow, adjusted for age.

Variables	Partial r	*p*	*df*
Years of arm exposure—right ulnar SNAP	0.004	0.991	9
Years of arm exposure—left ulnar SNAP	−0.167	0.623	9
Years of arm exposure—right ulnar NCV	−0.255	0.449	9
Years of arm exposure—left ulnar NCV	−0.236	0.485	9
**Right ulnar SNAP—left ulnar SNAP**	**0.688**	**0.019**	9
Right ulnar SNAP—right ulnar NCV	0.354	0.285	9
Right ulnar SNAP—left ulnar NCV	0.039	0.910	9
Left ulnar SNAP—right ulnar NCV	0.064	0.851	9
Left ulnar SNAP—left ulnar NCV	0.355	0.284	9
Right ulnar NCV—left ulnar NCV	0.564	0.071	9

Note: Values are Pearson partial correlation coefficients adjusted for patient age. SNAP, sensory nerve action potential recorded from digit 5; NCV, ulnar motor NCV across the elbow, recorded from the abductor digiti minimi muscle. Significant *p* values are shown in bold.

**Table 6:** Significant multiple regression models for the dependent variable SNAP (right hand).

Model	R	R Square	Adjusted R-Square	Standard Error of the Estimate
1	0.484 ^a^	0.235	0.222	12.976
2	0.670 ^b^	0.449	0.430	11.100

^a^. Predictors: (Constant), Patient age (years). ^b^. Predictors: (Constant), Patient age (years), Patient sex.

**Table 7:** Coefficients from the stepwise multiple linear regression models predicting right ulnar SNAP amplitude.

Stepwise Model	Predictor	B	SE	*β*	*t*	*p*
Step 1 Age only	Constant	59.500	6.774	—	8.783	<0.001
	Patient age (years)	−0.604	0.142	−0.484	−4.253	<0.001
Step 2 (final) Age + sex	Constant	60.030	5.796	—	10.358	<0.001
	Patient age (years)	−0.801	0.128	−0.642	−6.240	<0.001
	Patient sex	14.522	3.053	0.490	4.757	<0.001

Note: The dependent variable is RIGHT ulnar SNAP amplitude recorded from digit 5 (µV). Step 1 contains age only; Step 2 is the final model after patient sex was added by the Stepwise procedure. B, unstandardized regression coefficient; SE, standard error; β, standardised regression coefficient. SPSS values reported as 0.000 are presented as *p* < 0.001. The direction of the coefficient for patient sex depends on the coding used in the dataset. If female = 1 and male = 0, B = 14.522 indicates a 14.522 µV higher right SNAP amplitude in females than in males after adjustment for age.

**Table 8:** Stepwise multiple linear regression models predicting left ulnar SNAP amplitude.

Stepwise Model	Predictors Retained	R	R^2^	Adjusted R^2^	SEE
Step 1	Patient age	0.421	0.177	0.162	14.585
Step 2 (final)	Patient age + patient sex	0.670	0.449	0.428	12.048

Note: Step 1 contains patient age only. Step 2 is the final model after patient sex was added by the Stepwise procedure. R^2^ represents the proportion of variance explained by the model; adjusted R^2^ accounts for the number of predictors. SEE, standard error of the estimate. Candidate predictors not retained in the final model were years of arm exposure, dominant hand, and ulnar neuropathy at the elbow.

**Table 9:** Coefficients from the stepwise multiple linear regression models predicting left ulnar SNAP amplitude.

Stepwise Model	Predictor	B	SE	*β*	*t*	*p*
Step 1 Age only	Constant	53.018	7.737	—	6.853	<0.001
	Patient age (years)	−0.547	0.160	−0.421	−3.410	0.001
Step 2 (final) Age + sex	Constant	54.494	6.398	—	8.518	<0.001
	Patient age (years)	−0.804	0.142	−0.619	−5.676	<0.001
	Patient sex	17.899	3.501	0.558	5.112	<0.001

Note: The dependent variable is LEFT ulnar SNAP amplitude recorded from digit 5 (µV). Step 1 contains age only; Step 2 is the final model after patient sex was added by the Stepwise procedure. B, unstandardized regression coefficient; SE, standard error; β, standardised regression coefficient. SPSS values reported as 0.000 are presented as *p* < 0.001. The direction of the coefficient for patient sex depends on the coding used in the dataset. If female = 1 and male = 0, B = 17.899 indicates a 17.899 µV higher left SNAP amplitude in females than in males after adjustment for age.

**Table 10:** Multicollinearity diagnostics for predictors included in the logistic regression model.

Predictor	Tolerance	VIF
Years of arm exposure	0.428	2.334
Patient age (years)	0.292	3.430
Patient sex	0.516	1.937
Dominant hand	0.934	1.070
Number of comorbidities	0.848	1.179
RIGHT ulnar SNAP (D5) (µV)	0.357	2.798
LEFT ulnar SNAP (D5) (µV)	0.339	2.949

Note: Tolerance = 1/VIF. The values indicate no severe multicollinearity among the predictors. Age has the lowest tolerance (0.292) and the highest VIF (3.430), indicating the greatest, but still acceptable, overlap with the other predictors. VIF, variance inflation factor; SNAP, sensory nerve action potential recorded from digit 5. These diagnostics refer to the predictors entered into the logistic regression model with UNE as the outcome.

**Table 11:** Multiple linear regression coefficients for right ulnar SNAP amplitude.

Predictor	*B*	*SE*	*β*	*p*	Tolerance	VIF
Constant	69.105	13.027	—	<0.001	—	—
Patient sex	14.061	3.551	0.474	<0.001	0.689	1.452
Patient age (years)	−0.847	0.190	−0.679	<0.001	0.424	2.360
Dominant hand	−7.711	11.561	−0.067	0.508	0.975	1.026
Years of arm exposure	0.101	0.254	0.058	0.691	0.462	2.165
Ulnar neuropathy at the elbow	−1.807	3.490	−0.059	0.607	0.748	1.337

Note: The dependent variable is RIGHT ulnar SNAP amplitude recorded from digit 5 (µV). All predictors were entered simultaneously. B, unstandardized regression coefficient; SE, standard error; β, standardised regression coefficient; VIF, variance inflation factor. SPSS values reported as 0.000 are presented as *p* < 0.001.

**Table 12:** Multiple linear regression coefficients for left ulnar SNAP amplitude.

Predictor	*B*	*SE*	*β*	*p*	Tolerance	VIF
Constant	70.187	13.888	—	<0.001	—	—
Patient sex	14.947	3.915	0.466	<0.001	0.685	1.460
Patient age (years)	−0.741	0.209	−0.570	0.001	0.393	2.543
Dominant hand	−12.669	12.184	−0.106	0.303	0.976	1.025
Years of arm exposure	−0.086	0.277	−0.048	0.756	0.434	2.304
Ulnar neuropathy at the elbow	−6.447	3.781	−0.198	0.094	0.758	1.319

Note: The dependent variable is LEFT ulnar SNAP amplitude recorded from digit 5 (µV). All predictors were entered simultaneously. B, unstandardized regression coefficient; SE, standard error; β, standardised regression coefficient; VIF, variance inflation factor. SPSS values reported as 0.000 are presented as *p* < 0.001. The direction of the patient-sex coefficient depends on the coding used in the dataset.

**Table 13:** Multiple linear regression coefficients for right across-elbow ulnar NCV.

Predictor	*B*	*SE*	*β*	*p*	Tolerance	VIF
Constant	54.592	13.186	—	<0.001	—	—
Patient sex	1.942	6.812	0.048	0.777	0.912	1.096
Patient age (years)	−0.273	0.412	−0.163	0.512	0.432	2.316
Years of arm exposure	0.355	0.549	0.155	0.522	0.451	2.219

Note: The dependent variable is RIGHT ulnar motor nerve conduction velocity across the elbow (m/s), recorded from the abductor digiti minimi muscle. All predictors were entered simultaneously. B, unstandardized regression coefficient; SE, standard error; β, standardised regression coefficient; VIF, variance inflation factor. SPSS values reported as 0.000 are presented as *p* < 0.001.

**Table 14:** Multiple linear regression coefficients for left across-elbow ulnar NCV.

Predictor	*B*	*SE*	*β*	*p*	Tolerance	VIF
Constant	36.337	10.825	—	0.002	—	—
Patient sex	1.767	5.779	0.051	0.761	0.877	1.140
Patient age (years)	0.316	0.335	0.219	0.352	0.460	2.173
Years of arm exposure	−0.279	0.459	−0.138	0.547	0.481	2.080

Note: The dependent variable is LEFT ulnar motor nerve conduction velocity across the elbow (m/s), recorded from the abductor digiti minimi muscle. All predictors were entered simultaneously. B, unstandardized regression coefficient; SE, standard error; β, standardised regression coefficient; VIF, variance inflation factor. SPSS values reported as 0.000 are presented as *p* < 0.001.

**Table 15:** Variance explained by the multiple linear regression model for right ulnar SNAP amplitude.

Predictors Entered Simultaneously	R	R^2^	Adjusted R^2^	Standard Error of the Estimate
Patient age, patient sex, dominant hand, years of arm exposure, and ulnar neuropathy at the elbow	0.676	0.457	0.408	11.320

Note: The dependent variable is RIGHT ulnar SNAP amplitude recorded from digit 5 (µV). All predictors were entered simultaneously. R^2^, coefficient of determination; Adjusted R^2^, coefficient of determination adjusted for the number of predictors and sample size.

**Table 16:** Variance explained by the multiple linear regression model for left ulnar SNAP amplitude.

Predictors Entered Simultaneously	R	R^2^	Adjusted R^2^	Standard Error of the Estimate
Patient age, patient sex, dominant hand, years of arm exposure, and ulnar neuropathy at the elbow	0.700	0.491	0.440	11.927

Note: The dependent variable is LEFT ulnar SNAP amplitude recorded from digit 5 (µV). All predictors were entered simultaneously. R^2^, coefficient of determination; Adjusted R^2^, coefficient of determination adjusted for the number of predictors and sample size.

## Data Availability

The datasets analysed during the present study are not publicly available because they form part of an ongoing research project and contain potentially identifiable clinical information. However, they may be made available by the corresponding author upon reasonable request and subject to applicable ethical and data protection requirements.

## Abbreviations

The following abbreviations are used in this manuscript:

AANEM	American Association of Neuromuscular & Electrodiagnostic Medicine
CTS	carpal tunnel syndrome
CuTS	cubital tunnel syndrome
NCS	nerve conduction studies
NCV	nerve conduction velocity
SNAP	sensory nerve action potential
UNE	ulnar neuropathy at the elbow
